# Synergistic Effects of Hybrid Bio-Fillers and Modified Natural Rubber on Natural Rubber Composite Properties

**DOI:** 10.3390/polym17050632

**Published:** 2025-02-26

**Authors:** Supharat Inphonlek, Namthip Bureewong, Supawat Kotchapradit, Yupaporn Ruksakulpiwat, Chaiwat Ruksakulpiwat

**Affiliations:** 1 School of Polymer Engineering, Institute of Engineering, Suranaree University of Technology, Nakhon Ratchasima 30000, Thailand; supharat.inph@gmail.com (S.I.); namthipkhm@gmail.com (N.B.); 2 Research Center for Biocomposite Materials for Medical Industry and Agricultural and Food Industry, Suranaree University of Technology, Nakhon Ratchasima 30000, Thailand; 3 School of Electronic Engineering, Institute of Engineering, Suranaree University of Technology, Nakhon Ratchasima 30000, Thailand; supawat@sut.ac.th

**Keywords:** natural rubber composites, hybrid bio-fillers, rice husk silica, hydroxyapatite, modified natural rubber

## Abstract

This work aims to investigate the synergistic effects of hybrid bio-fillers and compatibilizers on the properties of natural rubber composites. Rice husk silica (RSi) and hydroxyapatite (HA), derived from rice husk ash and seabass fish scales, respectively, were successfully prepared and used as bio-fillers. Poly(acrylic acid-co-acrylamide)-grafted deproteinized natural rubber (gDPNR) was synthesized via emulsion graft copolymerization, achieving a grafting efficiency and grafting percentage of 15.94 and 4.23%, respectively. The gDPNR was utilized as a compatibilizer in the preparation of natural rubber composites. The addition of hybrid bio-fillers at an RSi-to-HA ratio of 25:75 exhibited superior mechanical properties compared to composites containing a single filler. The incorporation of gDPNR improved filler dispersion and interfacial adhesion between the NR matrix and the bio-fillers, further enhancing the mechanical, thermal, and dielectric properties. The composite with hybrid bio-fillers and 10 phr of gDPNR exhibited the highest tensile strength, showing a 2.10-fold and 1.06-fold improvement over neat natural rubber composite and hybrid filler composite without compatibilizer, respectively. The presence of polar functional groups in gDPNR enhanced the dielectric constant of the natural rubber composites. These composites could have potential in sustainable industrial applications, including flexible electronics and eco-friendly devices.

## 1. Introduction

The development of composite materials with enhanced mechanical, thermal, dielectric, and biocompatible properties has attracted considerable attention in diverse fields, including biomedical, structural, electronic, and environmental applications [1,2,3,4]. Natural rubber (NR), a versatile biopolymer, is widely utilized in industrial applications due to its excellent mechanical properties, elasticity, and resilience [5]. However, its inherent limitations, such as low thermal stability and susceptibility to aging, hinder its performance in advanced material applications. To overcome these drawbacks, the incorporation of reinforcing agents into NR has emerged as an effective strategy for enhancing composite properties [6].

Sustainable fillers have gained prominence as key components in the development of advanced NR composites, meeting the growing demand for environmentally friendly and sustainable materials. Derived from renewable, biodegradable, or waste resources, these fillers offer a green alternative to conventional synthetic fillers. Among the promising bio-based fillers, rice husk silica (RSi) from rice husk ash and hydroxyapatite (HA) from fish scales stand out. Rice husk silica, derived from agricultural waste, is an amorphous silica with a high surface area and low density, which enhances the mechanical properties, thermal stability, and durability of NR composites [7,8]. Similarly, hydroxyapatite, a calcium phosphate bioceramic sourced from fish scales, exhibits biocompatibility, rigidity, dielectric properties, and thermal stability [9,10,11]. Incorporating these fillers not only improves the performance of NR composites but also promotes waste valorization, contributing to environmental sustainability.

The incorporation of hybrid fillers, a combination of different reinforcing agents, offers synergistic effects that significantly enhance the properties of NR composites [12,13,14]. For instance, Qian et al. demonstrated the potential of natural hybrid fillers composed of biochar and silica in natural rubber composites [15]. This approach improved both mechanical and electrical properties, as evidenced by increased tensile strength and dielectric constant compared to a single-filler system. However, the hydrophobicity of NR often leads to poor compatibility with polar fillers, resulting in suboptimal filler dispersion and weak interfacial adhesion. To overcome this limitation, compatibilizers are employed to enhance the interactions between the NR matrix and fillers.

Compatibilizers play a critical role in improving interfacial bonding, thereby enhancing the overall properties of composites. These agents, such as silane coupling agents [16,17] and modified natural rubber derivatives [18,19], act as bridges by introducing functional groups that interact with both the non-polar NR matrix and the fillers. This enhanced adhesion facilitates better stress transfer, leading to improved mechanical properties. The grafted natural rubber derivatives, like poly(acrylic acid-co-acrylamide)-grafted deproteinized natural rubber (gDPNR), can be synthesized via emulsion graft copolymerization. The gDPNR features a unique dual structure: polar groups that enhance interactions with fillers and a non-polar backbone that ensures compatibility with the NR matrix. This dual functionality significantly improves composite performance. Furthermore, the polar components introduced by gDPNR provide a high dielectric constant, enabling its application in energy storage devices [20]. Therefore, gDPNR not only acts as an effective compatibilizer but also enhances the functional capabilities of NR composites.

This study aims to develop and characterize natural rubber composites reinforced with hybrid bio-fillers (rice husk silica and hydroxyapatite) and compatibilized with poly(acrylic acid-co-acrylamide)-grafted DPNR (gDPNR). Despite advancements in natural rubber composites, achieving strong interfacial adhesion and a well-balanced combination of mechanical, thermal, and dielectric properties remains a challenge. In this work, the incorporation of these sustainable fillers, along with gDPNR as a compatibilizer, is studied as a strategy to enhance the overall properties of the composites while promoting eco-friendly material solutions. The study investigates the synergistic effects of hybrid bio-fillers and compatibilizer on composite properties to understand their impact on composite performance. This work could contribute to the development of sustainable industrial materials, bio-based flexible electronics, and environmentally friendly engineering applications for high-performance and eco-conscious natural rubber composites.

## 2. Materials and Methods

### 2.1. Materials

Rice husk ash was obtained from Chia Meng Co., Ltd. (Nakhon Ratchasima, Thailand). Seabass fish scales were sourced from a local shop (Rayong, Thailand). Natural rubber latex, treated with high ammonia and a dry rubber content of 60 wt%, was purchased from Chemical and Materials Co., Ltd. (Bangkok, Thailand). Sodium dodecyl sulfate (SDS) and acrylamide (AM) were supplied by LOBA Chemie Pvt. Ltd. (Mumbai, India). Urea, hydrochloric acid (HCl), and toluene were provided by RCI Labscan Co., Ltd. (Bangkok, Thailand). Acrylic acid (AA), cumene hydroperoxide (CHP), and 1-[3-(Trimethoxysilyl)propyl]urea coupling agent (CA) were supplied by Aldrich (St. Louis, MO, USA). AA was passed through a column packed with alumina adsorbent before use. Sodium hydroxide (NaOH) and acetic acid (CH_3_COOH) were sourced from CARLO ERBA Reagents (Milan, Italy). Tetraethylene pentamine (TEPA) was supplied by Acros Organics (Geel, Antwerp, Belgium). Teric (10 wt%, N-cyclohexyl-2-benzothiazole sulfenamide (CBS), and tetramethylthiuram disulfide (TMTD) were purchased from the Rubber Authority of Thailand (Bangkok, Thailand). Ethanol was supplied by Duksan Reagents (Ansan, Gyeonggi, Reublic of Korea). Stearic acid (SA), zinc oxide (ZnO), and sulfur (S) were provided by Chemical Innovation Co., Ltd. (Bangkok, Thailand).

### 2.2. Preparation of Rice Husk Silica Particles

The rice husk silica (RSi) particles were prepared from rice husk ash using a precipitation method. First, the rice husk ash was leached with 1 M HCl solution at 90 °C under stirring with a magnetic stirrer for 3 h. The leached ash was then filtered, washed with deionized water until the pH was neutral, and dried in a hot-air oven at 110 °C for 12 h to obtain purified rice husk ash. Next, the purified rice husk ash was treated with 1 M NaOH solution at 90 °C for 12 h under continuous stirring to extract silica. The mixture was then filtered to obtain a clear sodium silicate solution. Subsequently, 1 M CH_3_COOH solution was added dropwise to the sodium silicate solution under mechanical stirring at room temperature until the pH was neutral, resulting in the formation of gel. The gel was washed with deionized water until its electrical conductivity was less than 6 µS/cm and was then dried using a freeze dryer to produce the RSi particles.

### 2.3. Preparation of Hydroxyapatite Particles

The hydroxyapatite (HA) particles were prepared from seabass fish scales following a previously reported method [21]. First, the fish scales were thoroughly washed with deionized water multiple times using a mechanical stirrer and were then dried in a hot-air oven at 80 °C for 24 h. To remove surface proteins, the dried fish scales were treated with 0.1 M HCl solution under stirring for 30 min at room temperature. The scales were then filtered, washed with deionized water until the pH was neutral, and dried again in a hot-air oven at 80 °C for 24 h. The fish scales were subjected to alkali heat treatment by immersing them in a 10%*w*/*v* NaOH solution. This treatment was conducted using an autoclave sterilizer at 121 °C for 30 min. After treatment, the slurry of fish scales was washed with deionized water until its electrical conductivity was less than 6 µS/cm. The washed slurry was then dried using a freeze-dryer to obtain a fine powder. The resulting products were collected for further characterization.

### 2.4. Characterization of Bio-Fillers

The chemical functional groups of rice husk silica and hydroxyapatite particles were analyzed using FTIR spectroscopy (TENSOR 27, Bruker, Billerica, MA, USA). The samples were mixed with potassium bromide using an agate mortar and pressed into pellets to prepare test specimens with smooth surfaces for transmittance measurements. The measurements were performed in the wavenumber range of 4000 to 400 cm^−1^, with a resolution of 4 cm^−1^ and 64 scans.

The crystalline phase compositions and diffraction patterns of rice husk silica and hydroxyapatite particles were analyzed using X-ray diffraction (XRD) (D2 PHASER, Bruker, Billerica, MA, USA). The analysis was conducted with a Cu Kα radiation source operating at 30 kV and 10 mA. The 2θ range for the measurement was set between 10° and 60°.

The morphology of rice husk silica and hydroxyapatite particles was analyzed using FESEM (AURIGA, Carl Zeiss, Oberkochen, Germany). The dried samples were dispersed in ethanol using an ultrasonic probe, and the resulting dispersion was dropped onto aluminum tape. The samples were left to dry overnight in a desiccator and were then coated with carbon prior to observation.

### 2.5. Preparation of Poly(Acrylic Acid-co-Acrylamide)-Grafted Deproteinized Natural Rubber

The deproteinized natural rubber (DPNR) latex was prepared according to the method described by Kawahara et al. [22]. In this process, natural rubber (NR) latex was incubated with 0.1 wt% urea and 1 wt% SDS. The impurities and proteins were removed by centrifugation. Subsequently, poly(acrylic acid-co-acrylamide)-grafted deproteinized natural rubber (gDPNR) was synthesized via emulsion graft copolymerization, based on a previous report with modifications [23]. DPNR latex and 5 phr of Teric16A were added into a reactor equipped with a mechanical stirrer. The mixture was purged with nitrogen gas for 45 min at 50 °C under continuous stirring at 100 rpm. Next, CHP, AA (with 40 mol% of AA neutralized using a 20 wt% NaOH solution), AM, and TEPA were introduced into the reactor. The weight ratio of CHP to TEPA was maintained at 1:1, while the comonomer ratio of AA to AM was set at 60:40 by weight. The total comonomer content was fixed at 15 phr, and the total solid content was maintained at 20 wt%. The polymerization reaction was carried out for 6 h. The resulting latex was poured into a tray and dried in a hot-air oven at 60 °C for 24 h.

### 2.6. Characterization of Poly(Acrylic Acid-co-Acrylamide)-Grafted Deproteinized Natural Rubber

The grafting efficiency and grafting percentage were determined using the gravimetric method. Dried samples were weighed and then immersed in deionized water for 72 h to remove any ungrafted products. The deionized water was replaced daily during this process. After extraction, the samples were dried at 60 °C for 24 h. The grafting efficiency and grafting percentage were calculated using the following equations [24]:

Grafting efficiency (%) = weight of (PAA-co-PAM) grafted × 100/weight of total polymer formed(1)

Grafting percentage (%) = weight of (PAA-co-PAM) grafted × 100/weight of DPNR used.(2)

Fourier transform infrared spectroscopy (FTIR) was employed to analyze the chemical structure of (PAA-co-PAM)-grafted DPNR using the attenuated total reflection (ATR) mode on a Tensor 27 FTIR spectrometer (Bruker, Billerica, MA, USA). The measurements were conducted with a resolution of 4 cm^−1^ over 64 scans. A background spectrum was recorded prior to sample analysis. The FTIR spectra of the samples were collected within the wavenumber range of 4000–400 cm^−1^.

### 2.7. Preparation of Natural Rubber Composites

**Figure 1:**
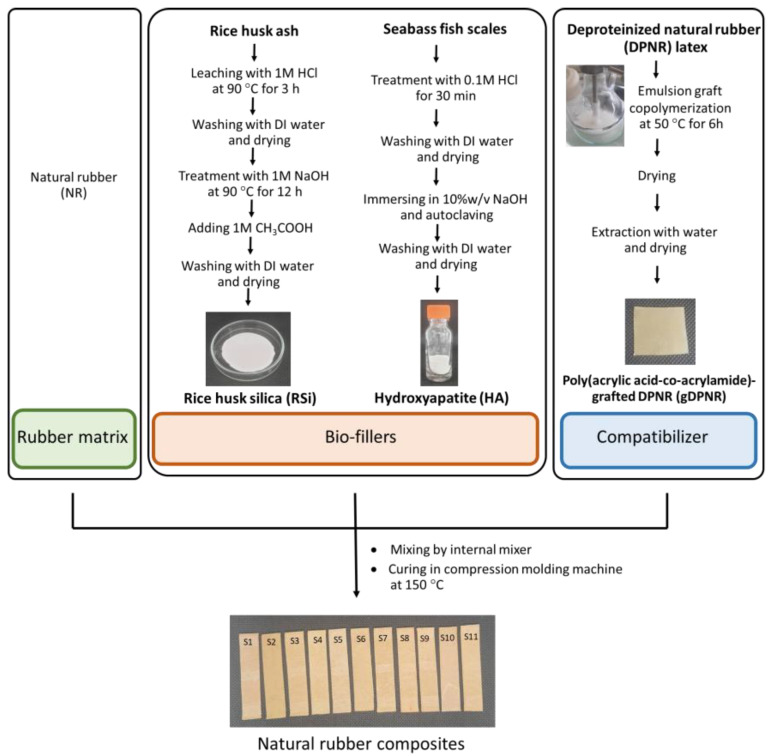
Schematic representation of preparation of natural rubber composites.

Natural rubber composites were prepared using an internal mixer operating at 60 °C and a roller speed of 40 rpm. The process began with the mastication of natural rubber for 3 min, followed by the sequential addition of activators (3 min), fillers (6 min), compatibilizers (2 min), and accelerators (3 min). Finally, the vulcanizing agent was incorporated and mixed for 3 min using a two-roll mill. The rubber compounds were cured in a compression molding machine at 150 °C, with the optimal cure time for each compound determined using a moving die rheometer (MDR 3000, MonTech, Buchen, Germany). The preparation process and sample compositions of the natural rubber composites are shown in Figure 1 and Table 1, respectively. Sample S1 represents the neat natural rubber composite. Samples S2–S6 consist of natural rubber composites incorporating varying proportions of rice husk silica and hydroxyapatite. Samples S7–S10 include natural rubber composites containing bio-fillers with different amounts of gDPNR used as a compatibilizer. For comparison, sample S11 includes bio-fillers with a coupling agent (CA) as the compatibilizer.

### 2.8. Characterization of Natural Rubber Composites

The cure characteristics, such as scorch time (T_s2_), cure time (T_c90_), minimum torque (ML), maximum torque (MH), delta torque (MH-ML), and cure rate index (CRI) of rubber compounds were determined using a moving die rheometer (MDR 3000, MonTech, Buchen, Germany) according to ASTM D2084 with a temperature at 150 °C.

The swelling degree of natural rubber composites, expressed as the percentage change in mass, was determined in accordance with ASTM D471. Specimens were immersed in toluene for 22 h. The swelling degree was calculated using the following equation:

swelling degree (%) = (M_2_ − M_1_) × 100/M_1_ (3)

where M_1_ is the initial mass of the specimen (g), and M_2_ is the mass of the swollen specimen after immersion (g).

The fracture surface morphology of natural rubber composites was examined using FESEM (AURIGA, Carl Zeiss, Oberkochen, Germany). The cross-sectional surfaces, obtained by breaking the samples in liquid nitrogen and after tensile testing, were analyzed. The samples were fixed on stubs and coated with gold before observation.

The tensile strength, elongation at break, modulus at 100% strain (M100), and modulus at 300% strain (M300) of natural rubber composites were measured in accordance with ASTM D412. The tear strength was evaluated following ASTM D624. Mechanical testing was conducted using a universal testing machine (UTM, Model: 5565, INSTRON, Norwood, MA, USA) equipped with a 5 kN load cell and operating at a crosshead speed of 500 mm/min.

The hardness of the natural rubber composites was measured using a hardness tester (HPE II, Bareiss, Oberdischingen, Germany) in accordance with ASTM D2240 and employing the Shore A test method.

The thermal decomposition of natural rubber composites was analyzed using a thermogravimetric analyzer (TGA, NETZSCH, Selb, Germany). Samples were placed in an alumina pan and heated from 50 °C to 600 °C under a nitrogen atmosphere. The measurement was conducted at a heating rate of 10 °C/min with a nitrogen gas flow rate of 20 mL/min.

The viscoelastic properties of natural rubber composites were analyzed using dynamic mechanical analysis (DMA) with a DMA850 instrument (TA Instruments, New Castle, DE, USA) in dual cantilever mode. Measurements were performed with a dynamic strain of 0.5% at a frequency of 1 Hz. The samples were tested over a temperature range of −80 °C to 100 °C with a heating rate of 3 °C/min.

The dielectric constant of natural rubber composites was measured using an impedance analyzer (Keysight E4294A, Agilent, Santa Clara, CA, USA). The test specimen was placed between the electrodes, ensuring complete contact to eliminate air gaps. All measurements were performed at room temperature.

## 3. Results and Discussion

### 3.1. Properties of Bio-Fillers

#### 3.1.1. FTIR Analysis

**Figure 2:**
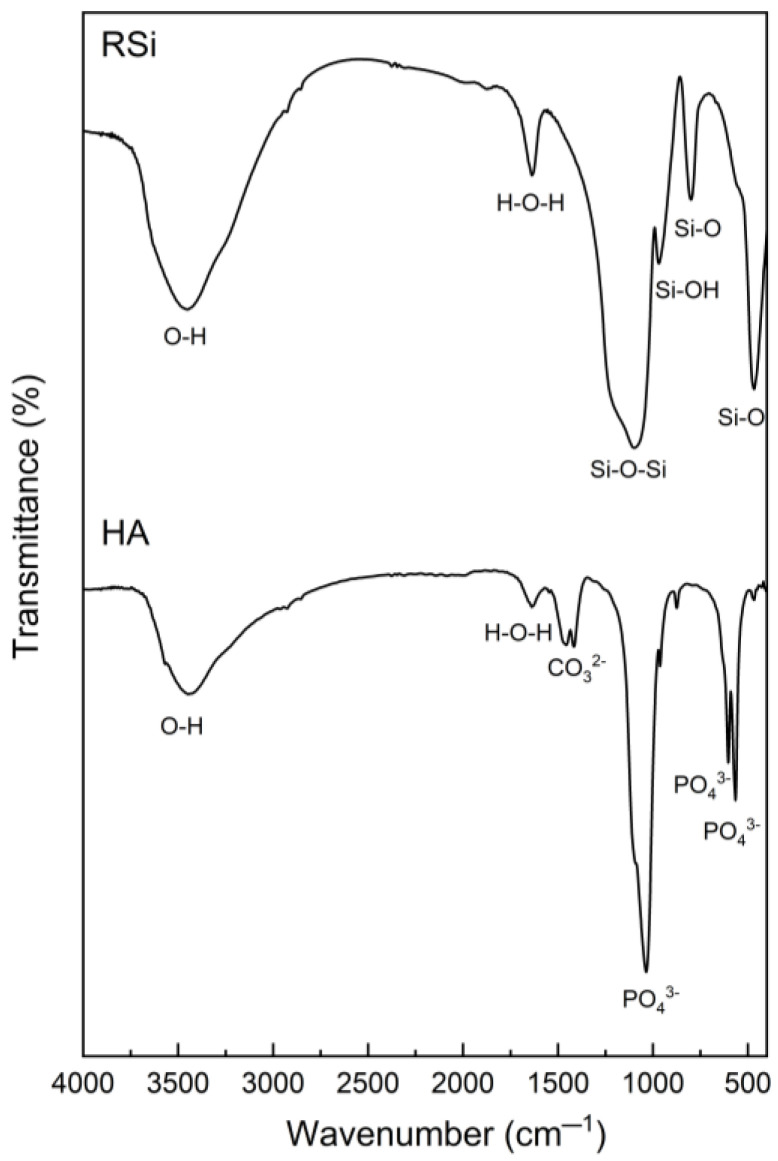
FTIR spectra of rice husk silica derived from rice husk ash and hydroxyapatite extracted from seabass fish scales.

Rice husk silica and hydroxyapatite were prepared from rice husk ash and seabass fish scales, respectively, and were utilized as bio-fillers in natural rubber composites. The FTIR spectra of these bio-fillers are shown in Figure 2, exhibiting their characteristic functional groups. The FTIR spectrum of rice husk silica displayed a broad peak at 3452 cm^−1^, corresponding to O-H stretching vibrations. A peak at 1637 cm^−1^ was attributed to H-O-H bending vibrations, associated with water molecules trapped within the silica structure. The prominent peak at 1098 cm^−1^ was assigned to Si-O-Si asymmetric stretching vibrations due to the characteristic of the silica network structure [25]. Additionally, a peak at 969 cm^−1^ was attributed to Si-OH bending vibrations, indicating the presence of surface silanol groups. Peaks at 799 and 467 cm^−1^ corresponded to Si-O symmetric stretching and Si-O bending, respectively [26,27,28]. For hydroxyapatite, the spectrum exhibited peaks at 3443 cm^−1^ and 1637 cm^−1^ corresponding to hydroxyl groups of adsorbed water. A sharp peak at 1035 cm^−1^ was attributed to the symmetric stretching of the phosphate (PO_4_^3^^−^) groups. Peaks at 603 and 566 cm^−1^ were associated with the asymmetric stretching and bending modes of phosphate groups, respectively. The peaks at 1455 and 1416 were attributed to asymmetric stretching of carbonate (CO_3_^2−^) groups, indicating carbonate substitution in the hydroxyapatite structure [29]. These results confirm the successful preparation of rice husk silica and hydroxyapatite with their functional groups, suitable for use as bio-fillers in composites.

#### 3.1.2. XRD Analysis

**Figure 3:**
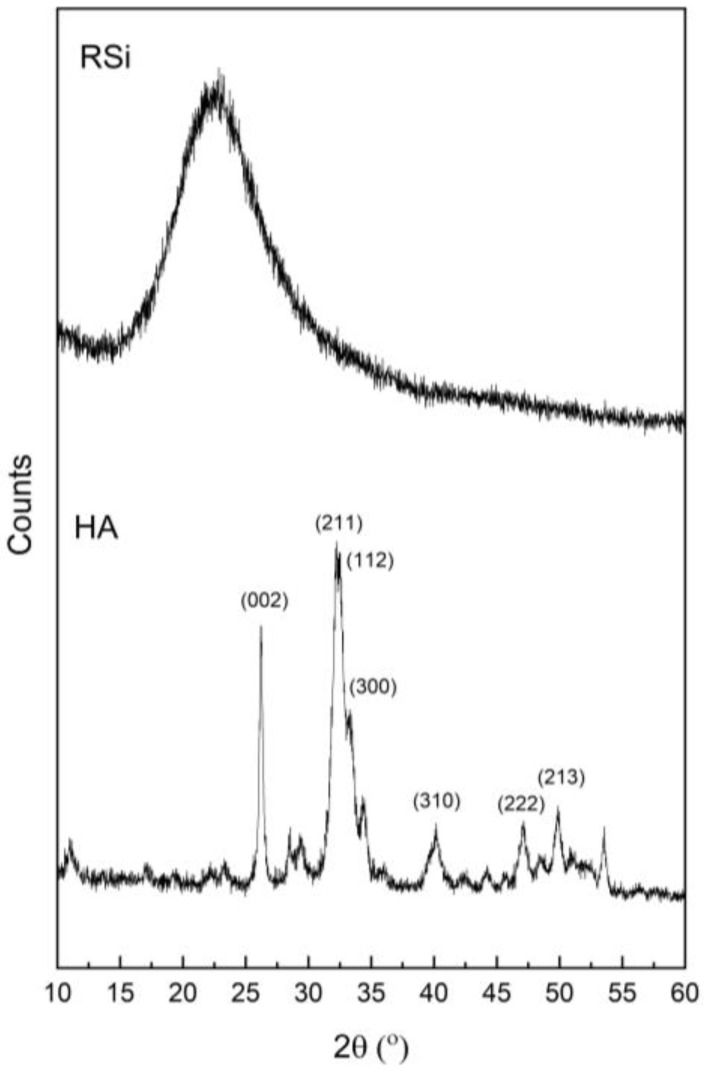
XRD patterns of rice husk silica derived from rice husk ash and hydroxyapatite extracted from seabass fish scales.

Figure 3 presents the XRD patterns of the prepared rice husk silica and hydroxyapatite. The XRD pattern of rice husk silica shows a broad peak at 2θ between 15° and 30°, which corresponds to the disordered arrangement of Si-O-Si bonds within the silica structure [30]. This broad peak indicates that the silica derived from rice husk ash exhibits an amorphous nature due to the relatively low calcination temperatures used during its preparation [31,32]. In contrast, the XRD pattern of hydroxyapatite displays sharp and well-defined peaks, signifying its high crystallinity. Diffraction peaks observed at 26.2°, 32.2°, 32.5°, 33.2°, 40.1°, 47.1°, and 49.8° correspond to the reflection planes (002), (211), (112), (300), (310), (222), and (213), respectively [33]. These peaks align well with the standard of hydroxyapatite pattern (JCPDS card No. 09-0432), confirming the successful synthesis of hydroxyapatite with a crystalline structure.

#### 3.1.3. Surface Morphology

**Figure 4:**
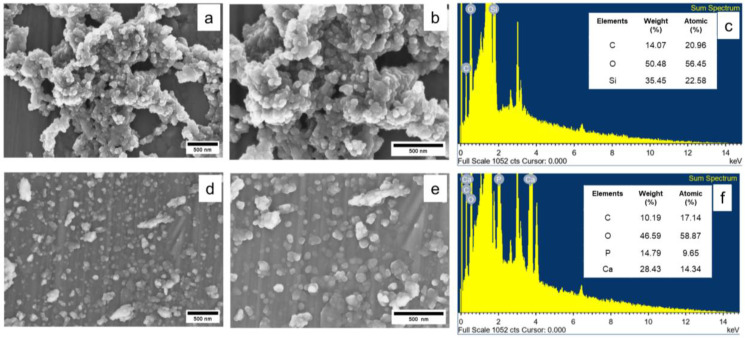
SEM images at ×30,000 and ×50,000 magnifications and EDS analysis of (**a**–**c**) rice husk silica derived from rice husk ash and (**d**–**f**) hydroxyapatite extracted from seabass fish scales.

Figure 4 shows SEM images of the prepared rice husk silica and hydroxyapatite particles. The rice husk silica exhibited a tendency to form agglomerates, appearing as clustered structures. The primary particle size of silica, determined using ImageJ software (IJ 1.46r image analyzer software), ranged from 27.91 to 33.49 nm. SEM-EDS analysis confirmed that the main elements present in rice husk silica were silicon (Si) and oxygen (O). In contrast, the hydroxyapatite particles exhibited an irregular shape. The particle size of hydroxyapatite was in the range of 41.31 to 85.16 nm. SEM-EDS analysis revealed the presence of carbon (C), oxygen (O), phosphorus (P), and calcium (Ca) in the structure. The addition of these bio-fillers in the natural rubber composites may influence the filler distribution, interactions, and overall properties of the composites.

### 3.2. Properties of Poly(Acrylic Acid-co-Acrylamide)-Grafted DPNR

**Figure 5:**
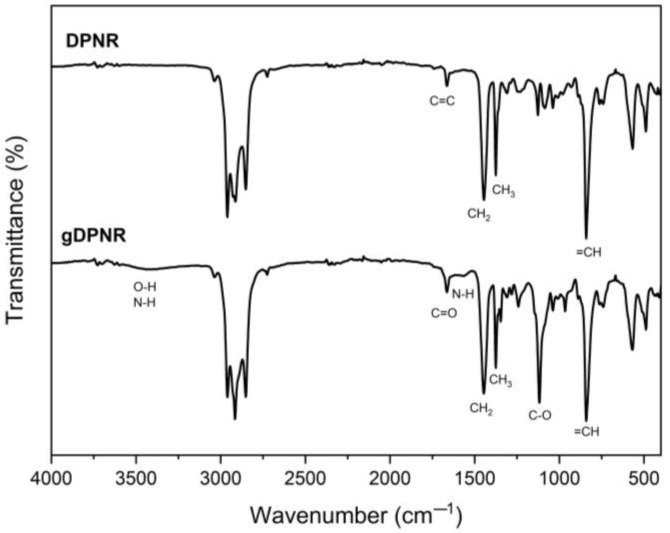
FTIR spectra of DPNR and poly(acrylic acid-co-acrylamide)-grafted DPNR (gDPNR).

The deproteinized natural rubber was modified through emulsion graft copolymerization with acrylic acid and acrylamide to produce poly(acrylic acid-co-acrylamide)-grafted DPNR (gDPNR). The grafting efficiency and grafting percentage of gDPNR were determined to be 15.94% and 4.23%, respectively. To confirm the successful grafting of poly(acrylic acid-co-acrylamide) (PAA-co-PAM) onto DPNR, FTIR spectroscopy was employed. The modified product was extracted with water to remove ungrafted materials, and its FTIR spectrum was compared with that of unmodified DPNR, as shown in Figure 5. The FTIR spectrum of unmodified DPNR exhibited characteristic peaks corresponding to the molecular structure of cis-1,4-polyisoprene, the primary polymer component of natural rubber. The observed characteristic peaks included those at 2990 cm^−1^ (C-H stretching), 1663 cm^−1^ (C=C stretching), 1446 cm^−1^ (CH₂ bending), 1375 cm^−1^ (CH_3_ bending), and 840 cm^−1^ (=CH out-of-plane bending) [34,35]. The FTIR spectrum of gDPNR retained these characteristic peaks of natural rubber, indicating the presence of the polyisoprene backbone. Additionally, new peaks were observed at 3200–3500 cm^−1^ (O-H and N-H stretching), 1664 cm^−1^ (C=O stretching), 1568 cm^−1^ (N-H bending), and 1118 cm^−1^ (C-O stretching). The appearance of these new peaks confirmed the successful grafting of PAA-co-PAM onto the DPNR [36,37]. As a result, the gDPNR exhibited functional groups capable of interacting with both natural rubber and polar fillers, demonstrating its potential as a compatibilizer for the development of advanced natural rubber composites.

### 3.3. Properties of Natural Rubber Composites with Bio-Fillers and Compatibilizers

#### 3.3.1. Cure Characteristics

Natural rubber composites were prepared by compounding natural rubber with various ingredients, including bio-fillers (rice husk silica and hydroxyapatite) and compatibilizers (gDPNR and coupling agent), in different formulations. The cure characteristics of these composites, as analyzed using a moving die rheometer (MDR), are summarized in Table 2. The addition of bio-fillers (samples S2–S6) resulted in a reduction in scorch time (T_s2_) and cure time (T_c90_) compared to the neat sample (sample S1). This decrease can be attributed to the catalytic nature of the polar bio-fillers, which enhance the thermal conductivity of the rubber compounds. Improved thermal conductivity facilitated more efficient heat transfer during vulcanization, thereby accelerating the initiation and completion of the vulcanization process [38]. As a result, these composites exhibited a higher cure rate index. The minimum torque (ML) values of samples S2–S6 were higher than that of sample S1, attributed to the increased viscosity of the uncured rubber caused by filler loading [39]. Among the composites, those with hydroxyapatite-rich conditions showed higher delta torque (MH−ML), indicating increased stiffness of the vulcanized rubber. Incorporation of compatibilizers, either gDPNR or coupling agent (CA), in the composites (samples S7–S11) further reduced scorch time and cure time while increasing delta torque compared to composites without compatibilizers (sample S5). The use of compatibilizers promoted better filler dispersion and enhanced matrix–filler interactions. This improvement resulted in faster curing, a higher vulcanization rate, and enhanced stiffness of the composites.

#### 3.3.2. Swelling Degree

**Figure 6:**
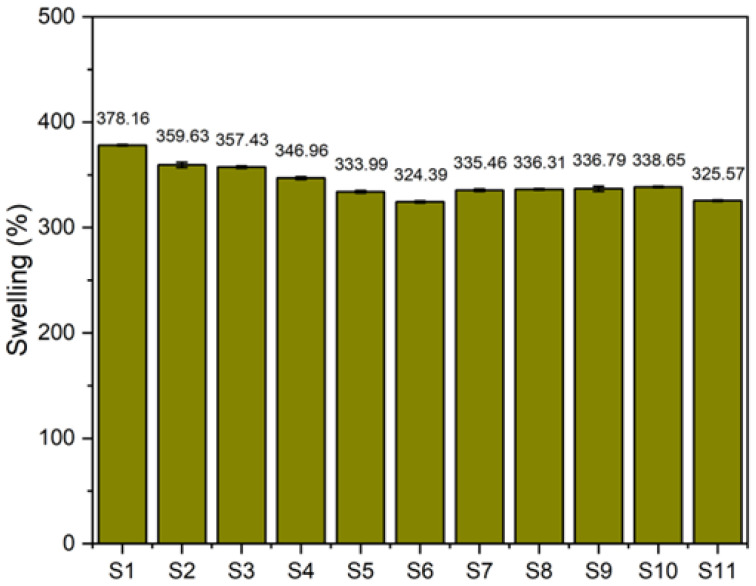
Swelling degree of natural rubber composites with various formulations.

Figure 6 illustrates the swelling degree of sulfur-cured natural rubber composites after immersion in toluene. The natural rubber without fillers exhibited the highest swelling degree at 378.16 ± 0.49% due to its lower crosslink density, which allowed greater solvent absorption. The incorporation of fillers resulted in a reduction in the swelling degree, which ranged from 324.39 ± 0.86% to 359.63 ± 2.52%. This reduction was attributed to the fillers restricting the free volume within the rubber matrix, thereby limiting solvent penetration [40]. Fillers act as physical barriers within the matrix, enhancing structural rigidity and reducing the permeability of the composites. Upon adding gDPNR as a compatibilizer, the swelling degree increased slightly, ranging from 335.46 ± 1.17% to 338.65 ± 0.57%, compared to the filler-containing composite without compatibilizer (333.99 ± 1.17%). This slight increase may result from the gDPNR being inserted between the chains of the natural rubber matrix, enhancing the flexibility of the polymer matrix and creating pathways that allow solvent absorption. However, the use of a coupling agent reduced the swelling degree to 325.57 ± 0.44%. This reduction is attributed to the improved filler–matrix bonding and enhanced filler dispersion achieved with the coupling agent. The coupling agent strengthens the network structure of the composite, minimizing free volume and restricting solvent absorption.

#### 3.3.3. Morphological Analysis of Fracture Surface

**Figure 7:**
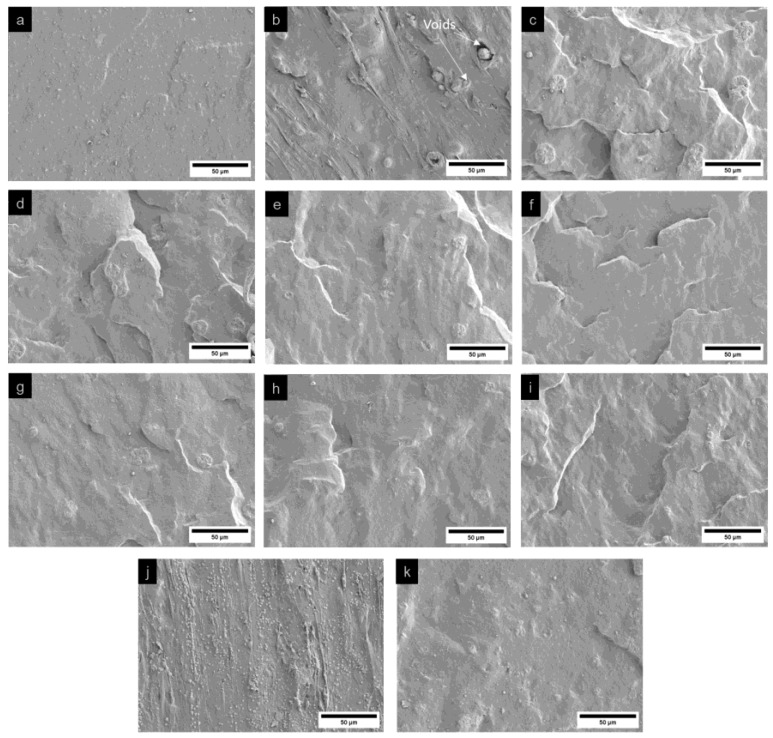
SEM images of the fracture surfaces of (**a**) sample 1, (**b**–**f**) samples 2–6, (**g**–**j**) samples 7–10, and (**k**) sample 11, fractured in liquid nitrogen.

**Figure 8:**
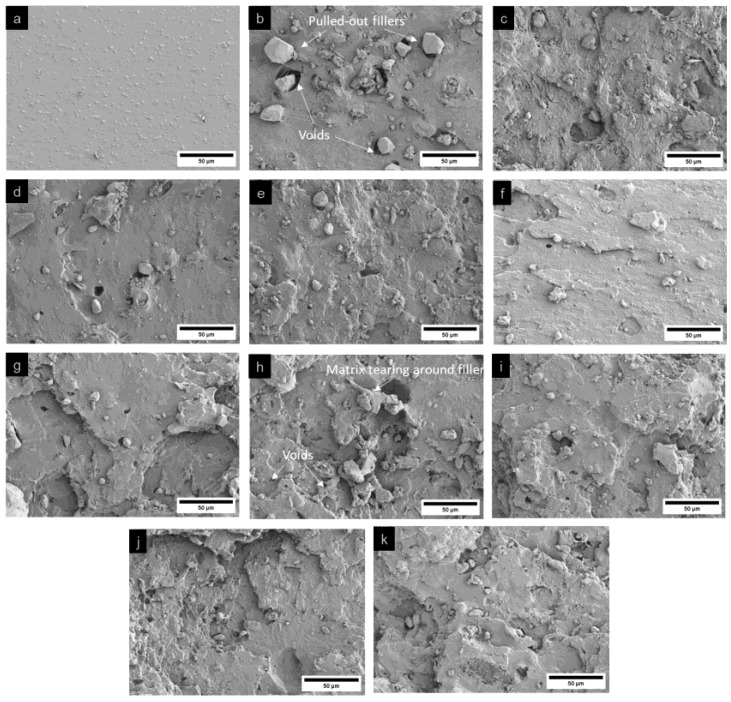
SEM images of the fracture surfaces of (**a**) sample 1, (**b**–**f**) samples 2–6, (**g**–**j**) samples 7–10, and (**k**) sample 11, subjected to tensile testing.

**Figure 9:**
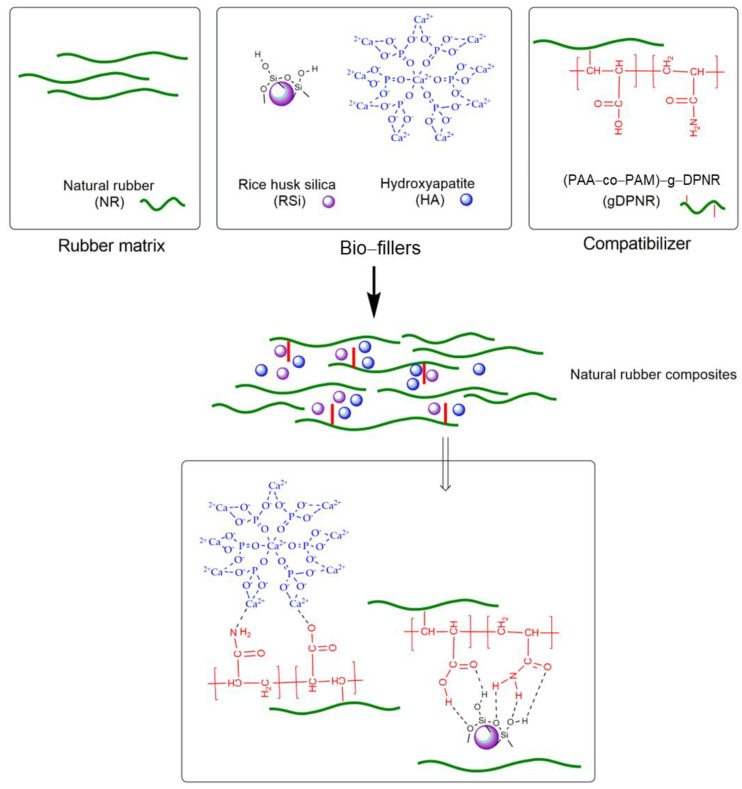
Schematic representation of the interactions of bio-fillers and gDPNR in the natural rubber composites.

The fracture morphology of natural rubber composites with bio-fillers and compatibilizers was analyzed using scanning electron microscopy (SEM) to evaluate the effects of these additives on fracture behavior within the rubber matrix. Fracture surfaces were examined for samples fractured in liquid nitrogen and those subjected to tensile testing, as shown in Figure 7 and Figure 8, respectively. For samples fractured in liquid nitrogen, brittle fracture was predominant, resulting in smooth and flat fracture surfaces compared to those subjected to tensile testing. The neat natural rubber (sample S1) exhibited a relatively smooth fracture surface, consistent with the absence of fillers. However, the incorporation of bio-fillers (rice husk silica and hydroxyapatite) resulted in rougher fracture surfaces. Samples subjected to tensile testing exhibited more pronounced roughness due to matrix deformation and energy dissipation during the applied loading. Among the natural rubber composites with bio-fillers (samples S2–S6), rice husk silica-rich samples showed larger voids with smooth void walls, caused by filler pull-out. These features indicate poor adhesion between the rice husk silica particles and the natural rubber matrix, attributed to the agglomeration of silica particles. The filler–filler interactions reduced their dispersion in the non-polar natural rubber matrix, leading to weak filler–matrix bonding and larger voids. The addition of compatibilizers, such as gDPNR and coupling agents, significantly improved the fracture morphology. In composites containing compatibilizers (samples S7–S11), the fracture surfaces were notably rougher with smaller voids. Evidence of matrix tearing around filler particles suggested enhanced matrix–filler interactions [41]. The gDPNR was particularly effective in improving filler dispersion and adhesion [42]. Its chemical structure, which includes both polar and non-polar functional groups, facilitated interactions with both the fillers and the natural rubber matrix. Their interactions are illustrated in Figure 9. The polar groups interacted with the surfaces of rice husk silica and hydroxyapatite, enhancing adhesion, while the non-polar groups ensured compatibility with the rubber matrix. This dual interaction mechanism contributed to better dispersion and stronger bonding, leading to improved fracture resistance of the composites.

#### 3.3.4. Mechanical Properties

**Figure 10:**
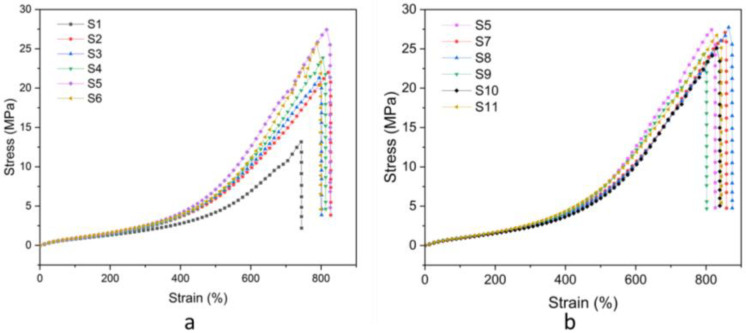
Stress–strain curves of (**a**) samples S1–S6 and (**b**) samples S5, S7–S11.

**Figure 11:**
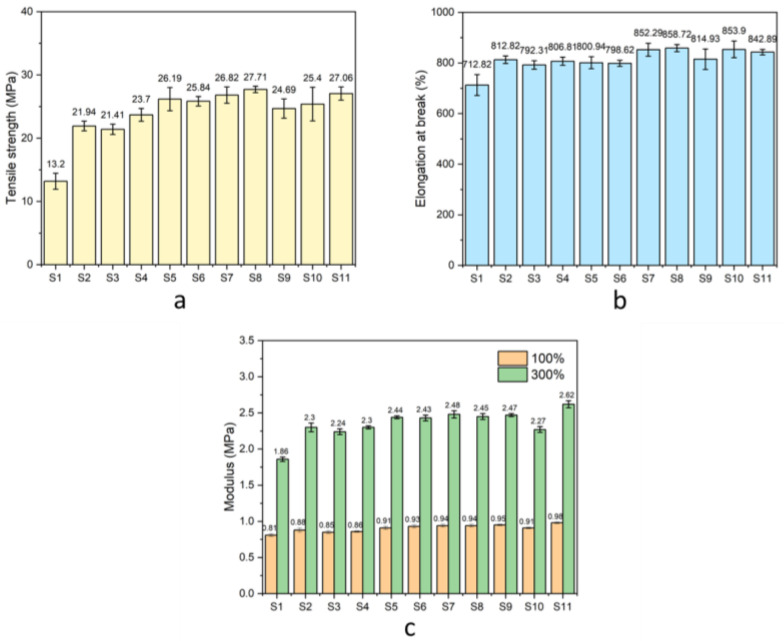
The (**a**) tensile strength, (**b**) elongation at break, and (**c**) modulus at 100 and 300% strain of the natural rubber composites.

The mechanical properties of the natural rubber composites were evaluated through tensile testing. Figure 10a,b displays the stress–strain curves of natural rubber composites with different formulations. The results for tensile strength, elongation at break, and modulus at 100% and 300% strain are shown in Figure 11a–c, respectively. The neat natural rubber (sample S1) showed a low tensile strength of 13.20 ± 1.27 MPa. Incorporating bio-fillers, including rice husk silica and hydroxyapatite in varying ratios, significantly enhanced the tensile strength due to the reinforcing effect of the fillers. Among these, the composite with a rice husk silica-to-hydroxyapatite ratio of 25:75 (sample S5) achieved the highest tensile strength of 26.19 ± 1.83 MPa, outperforming composites with a single filler. The improved mechanical performance can be attributed to the complementary properties of the fillers [15]. For the rice husk silica, SEM images revealed that it tended to agglomerate, resulting in uneven distribution within the matrix. This non-uniform dispersion reduced rubber–filler interaction and limited reinforcement [43]. Conversely, crystalline hydroxyapatite provided better rigidity and mechanical support, enhancing tensile strength. Adding compatibilizers, such as grafted DPNR (gDPNR) and a coupling agent (CA), further improved tensile strength by promoting better filler dispersion and stronger interfacial bonding with the natural rubber matrix. Specifically, the inclusion of 5 phr and 10 phr of gDPNR increased the tensile strength to 26.82 ± 1.29 MPa and 27.71 ± 0.50 MPa, respectively. However, at 20 phr, tensile strength decreased due to excessive compatibilizer disrupting filler dispersion and matrix integrity. Sample S8, with 10 phr of gDPNR, exhibited the highest tensile strength, showing a 2.10-fold improvement over the neat natural rubber (sample S1) and a 1.06-fold improvement over the hybrid filler composite without compatibilizer (sample S5). Notably, the tensile strength of sample S8 was comparable to that of S11, which used CA as a compatibilizer. Regarding elongation at break, composites with bio-fillers (samples S2–S6) demonstrated higher elongation than the unfilled natural rubber (sample S1). This improvement can be attributed to the moderate filler content, which did not overly restrict polymer chain mobility, thus maintaining the matrix’s flexibility. Adding gDPNR further enhanced elongation at break due to improved adhesion between the filler and matrix, which reduced failure points and allowed for greater material deformation before fracture [44]. The incorporation of bio-fillers significantly increased the modulus at 100% and 300% strain, as the fillers acted as reinforcing agents, restricting deformation in the rubber matrix. The addition of gDPNR further improved the modulus, indicating enhanced stiffness and mechanical properties of the natural rubber composites due to better filler–matrix compatibility. These results suggest that gDPNR serves as an effective compatibilizer to enhance the mechanical performance of natural rubber composites.

**Figure 12:**
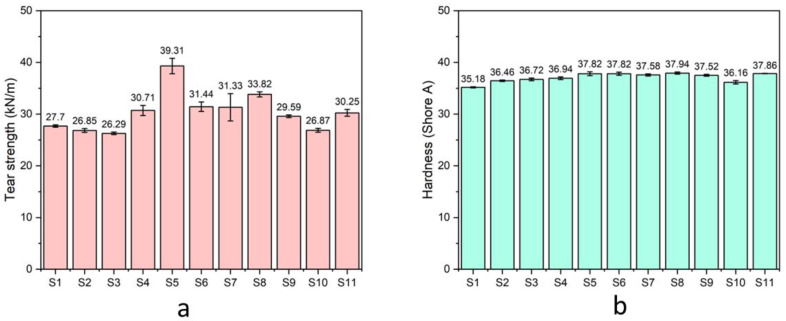
The (**a**) tear strength and (**b**) hardness of the natural rubber composites.

Figure 12a illustrates the tear strength of the prepared natural rubber composites. The composites containing rice husk silica-rich condition exhibited reduced tear strength, primarily due to the agglomeration of silica particles, which hindered effective dispersion within the natural rubber matrix. However, the addition of hybrid fillers (a combination of rice husk silica and hydroxyapatite) improved the tear strength of the composites. This enhancement can be attributed to the synergistic reinforcing effects of hybrid fillers. Proper dispersion of the fillers minimized agglomeration, providing a more uniform reinforcement that reduced weak points where cracks could initiate. This improved reinforcement enhanced the composite’s resistance to tear propagation, a critical property for rubber materials subjected to stress. Tear strength measures a material’s ability to resist the propagation of cracks or tears under mechanical stress. After adding compatibilizer, tear strength was decreased. However, composites containing gDPNR demonstrated higher tear strength compared to those with coupling agents. Figure 12b depicts the hardness of the natural rubber composites with various formulations. The inclusion of fillers and compatibilizers led to an increase in the hardness of the composites. Among the samples, the natural rubber composite containing hybrid bio-fillers and 10 phr of gDPNR exhibited the highest hardness, measured at 37.94 ± 0.21. This improved dispersion and strengthened matrix–filler interactions resulted in a more uniform stress distribution throughout the composites, further contributing to the observed increase in hardness [45].

#### 3.3.5. Thermal Properties

**Figure 13:**
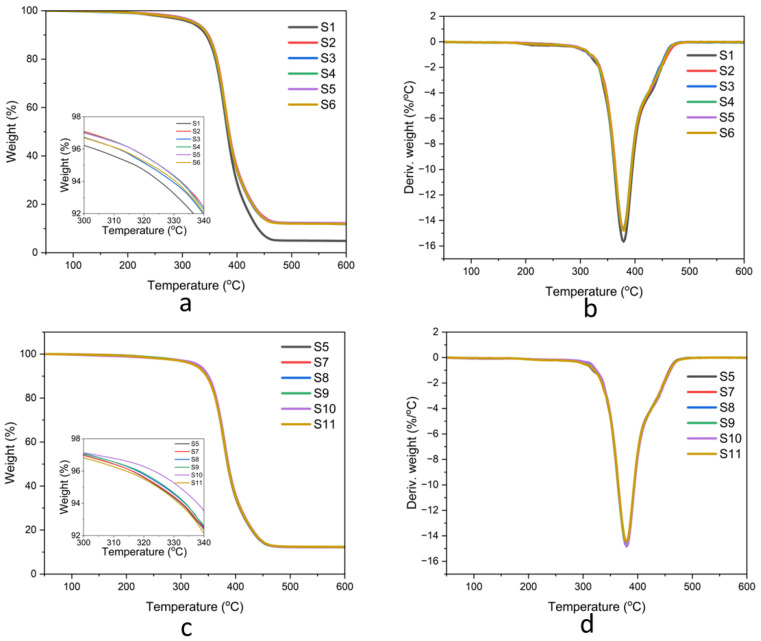
TGA and DTG thermograms of natural rubber composites: (**a**,**b**) samples S1–S6 and (**c**,**d**) samples S5, S7–S11.

The weight loss and first derivative curves of natural rubber composites are shown in Figure 13, and the thermal parameters, including the temperature at 5% weight loss (*T*_5_), the temperature at the maximum weight loss rate (*T_max_*), and the residue content, are summarized in Table 3. For neat natural rubber (sample S1), thermal degradation occurred in a single step, with *T*_5_ and *T_max_* values of 316.68 °C and 379.18 °C, respectively. The incorporation of bio-fillers (samples S2–S6) increased the *T*_5_ values to approximately 321.81–324.71 °C. This improvement in *T*_5_ is attributed to the presence of fillers, which restrict the mobility of natural rubber chains and create a barrier that delays the onset of thermal degradation. The addition of gDPNR as a compatibilizer further improved the thermal stability of the composites. The enhanced matrix–filler adhesion facilitated by gDPNR likely contributed to this improvement, as the compatibilizer promotes better interaction between the filler particles and the natural rubber matrix. For example, sample S8, which contained 10 phr of gDPNR, exhibited a *T*_5_ value of 327.10 °C. The results were consistent with those reported in the literature, where modified natural rubber was used as a compatibilizer [46]. Comparatively, composites containing gDPNR demonstrated higher *T*_5_ values than those using a coupling agent, emphasizing the effectiveness of gDPNR in delaying the onset of thermal degradation. Despite the differences in *T*_5_, the *T_max_* values for all composites remained similar to that of neat natural rubber, regardless of the presence of fillers or compatibilizers. This observation indicates that while fillers and compatibilizers effectively delay the initial degradation stage (*T*_5_), they do not alter the overall thermal degradation behavior (*T_max_*) of the natural rubber matrix.

#### 3.3.6. Dynamic Mechanical Properties

**Figure 14:**
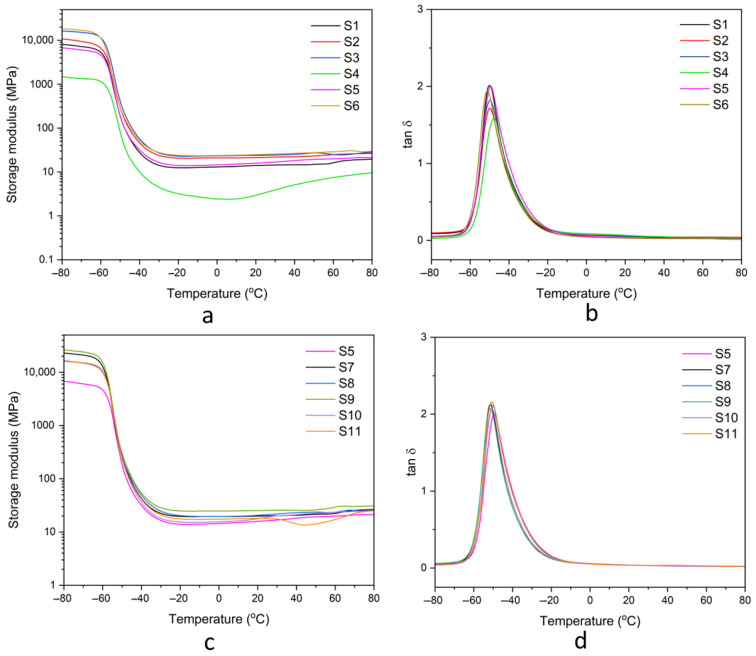
Storage modulus and damping factor of natural rubber composites: (**a**,**b**) samples S1–S6 and (**c**,**d**) samples S5, S7–S11.

The viscoelastic behavior of natural rubber composites was evaluated using dynamic mechanical analysis (DMA). The storage modulus (*E’*) and damping factor (tan δ) as functions of temperature are shown in Figure 14. At low temperatures, all composites exhibited a high storage modulus due to the tightly packed polymer chains with limited mobility in the glassy state. As the temperature increased, the storage modulus decreased, reflecting the material’s transition from a glassy state to a rubbery state, driven by increased molecular mobility [47]. The glass transition temperature (Tg) was determined from the peak of the tan δ curve, with the Tg values summarized in Table 4. Composites containing fillers exhibited a higher Tg compared to the unfilled sample. This increase is attributed to the restricted chain mobility caused by the fillers, which impede the segmental motion of the polymer chains. The addition of compatibilizers improved the interaction between the rubber matrix and the fillers, which reduced the rigidity of filler domains and enhanced the overall compatibility. The chemical structure of gDPNR, containing a non-polar natural rubber backbone, facilitated its integration into the natural rubber matrix. This integration reduced the chain restrictions of the matrix, thereby increasing flexibility. As a result, composites containing compatibilizers, particularly gDPNR, exhibited a lower glass transition temperature (Tg) compared to composites without compatibilizers. Furthermore, the incorporation of hybrid bio-fillers and gDPNR influenced the wet grip and rolling resistance of natural rubber composites, as observed from tan δ values at different temperatures (0 °C and 60 °C) [48]. Sample S8, which exhibited enhanced mechanical and thermal properties, also showed a higher tan δ at 0 °C compared to neat NR composite. A higher tan δ at 0 °C suggests improved wet traction by increasing friction under wet conditions. Additionally, it exhibited a low tan δ at 60 °C, which is advantageous for reducing rolling resistance. A lower tan δ at elevated temperature demonstrates reduced energy dissipation during deformation, contributing to lower energy consumption. These indicate the potential of the composite for sustainable automotive applications and energy-efficient material solutions.

#### 3.3.7. Dielectric Properties

**Figure 15:**
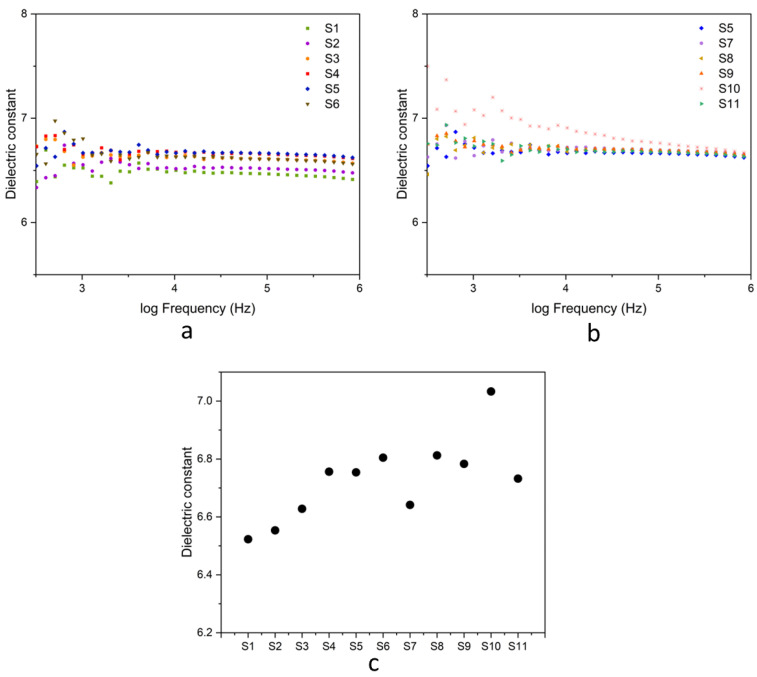
Dielectric constant of natural rubber composites: (**a**) samples S1–S6; (**b**) samples S5, S7–S11; (**c**) measurements at 1 kHz.

Figure 15a,b presents the dielectric constant of natural rubber composites as a function of frequency. At low frequencies, the composites exhibited a high dielectric constant. This behavior is attributed to the material having sufficient time to align with the applied electric field, leading to increased polarization. As the frequency increased, the dielectric constant decreased. This reduction is due to the inability of polarization mechanisms, such as dipolar and interfacial polarization, to respond the rapidly oscillating electric field. Natural rubber, with its non-polar structure, displayed a relatively low dielectric constant [49]. Figure 15c shows the comparison of dielectric constants of different composite types measured at 1 kHz. The addition of fillers increased the dielectric constant of the composites by introducing polar and interfacial polarization mechanisms. Among the composites, those containing hybrid fillers (a combination of rice husk silica and hydroxyapatite) exhibited a higher dielectric constant compared to composites with only silica. This result was consistent with the findings of Sundarabharathi et al., which demonstrated that hydroxyapatite, as a dielectric material, enhanced the dielectric properties of composites [50]. Furthermore, the incorporation of gDPNR as a compatibilizer improved the dielectric constant of the composites. The polar functional groups present in gDPNR actively respond to the applied electric field, enhancing polarization and contributing to the increase in the dielectric constant. These improvements underscore the potential of natural rubber composites for advanced applications, particularly in the development of flexible electronic devices and bio-based energy storage. These applications demand materials with excellent dielectric properties along with mechanical performance, making the optimized composites promising materials for development.

## 4. Conclusions

The rice husk silica (RSi) and hydroxyapatite (HA) were successfully derived from rice husk ash and seabass fish scales, respectively. Their physicochemical properties, including chemical functional groups, crystal structure, and morphology, were analyzed. Poly(acrylic acid-co-acrylamide)-grafted deproteinized natural rubber (gDPNR) was synthesized via emulsion graft copolymerization with a grafting efficiency of 15.94% and a grafting percentage of 4.23%. FTIR analysis confirmed the successful grafting process. Natural rubber composites were developed using RSi and HA as hybrid bio-fillers and gDPNR as a compatibilizer. The incorporation of hybrid bio-fillers, particularly at a RSi-to-HA ratio of 25:75, significantly enhanced the mechanical properties compared to single-filler systems. The addition of gDPNR further improved the mechanical and thermal properties of the composites. The composite containing hybrid bio-fillers and 10 phr of gDPNR exhibited the highest tensile strength and delayed the onset of thermal degradation, contributing to improved composite stability. The backbone of gDPNR provided compatibility with the natural rubber matrix, while its polar functional groups enhanced interactions with the fillers. This improved interaction between rubber matrix and fillers led to better overall composite performance. Additionally, the polar groups in gDPNR increased polarization, thereby enhancing the dielectric constant of the composites. The synergistic effects of hybrid bio-fillers and gDPNR have potential for the development of high-performance, sustainable rubber composites, making them suitable for a wide range of industrial applications.

## Figures and Tables

**Table 1:** Formulations of the prepared natural rubber composites.

Samples	Content (phr *)										
	S1	S2	S3	S4	S5	S6	S7	S8	S9	S10	S11
NR	100	100	100	100	100	100	95	90	80	-	100
gDPNR	-	-	-	-	-	-	5	10	20	100	-
CA	-	-	-	-	-	-	-	-	-	-	1
RSi	-	10	7.5	5.0	2.5	−	2.5	2.5	2.5	2.5	2.5
HA	-	-	2.5	5.0	7.5	10	7.5	7.5	7.5	7.5	7.5
SA	2	2	2	2	2	2	2	2	2	2	2
ZnO	4	4	4	4	4	4	4	4	4	4	4
CBS	2	2	2	2	2	2	2	2	2	2	2
TMTD	0.1	0.1	0.1	0.1	0.1	0.1	0.1	0.1	0.1	0.1	0.1
S	1	1	1	1	1	1	1	1	1	1	1

* phr is part per hundred of rubber.

**Table 2:** Cure characteristics of natural rubber composites at a curing temperature of 150 °C.

Samples	Materials	T_S2_ (min)	T_c90_ (min)	ML (dNm)	MH (dNm)	MH-ML (dNm)	CRI (min^−1^)
S1	NR	3.86	6.90	0.81	10.48	9.67	32.89
S2	NR/RSi10	2.29	4.03	1.72	10.85	9.13	57.47
S3	NR/RSi7.5-HA2.5	2.44	3.99	1.63	10.92	9.29	64.51
S4	NR/RSi5.0-HA5.0	2.69	4.15	1.48	11.18	9.70	68.49
S5	NR/RSi2.5-HA7.5	2.89	4.50	1.34	11.83	10.49	60.97
S6	NR/HA10	2.90	4.76	1.03	11.99	10.96	53.76
S7	NR/RSi2.5-HA7.5/gDPNR5	2.75	4.40	1.32	11.81	10.49	60.60
S8	NR/RSi2.5-HA7.5/gDPNR10	2.80	4.42	1.27	11.81	10.54	61.72
S9	NR/RSi2.5-HA7.5/gDPNR20	2.43	3.80	1.17	11.78	10.61	72.99
S10	gDPNR/RSi2.5-HA7.5	1.42	2.87	0.89	11.50	10.61	68.96
S11	NR/RSi2.5-HA7.5/CA1	2.55	4.16	1.30	12.14	10.84	62.11

**Table 3:** Thermal degradation temperatures and residue contents from TGA analysis of the prepared natural rubber composites.

Samples	Materials	*T*_5_ (min)	*T_max_* (min)	Residue at 600 °C (%)
S1	NR	316.68	379.18	4.85
S2	NR/RSi10	324.72	377.22	12.24
S3	NR/RSi7.5-HA2.5	321.81	379.31	11.79
S4	NR/RSi5.0-HA5.0	324.67	379.67	12.17
S5	NR/RSi2.5-HA7.5	324.46	379.46	12.27
S6	NR/HA10	322.14	379.64	11.93
S7	NR/RSi2.5-HA7.5/gDPNR5	324.57	379.57	12.23
S8	NR/RSi2.5-HA7.5/gDPNR10	327.10	379.60	12.15
S9	NR/RSi2.5-HA7.5/gDPNR20	327.03	379.53	12.38
S10	gDPNR/RSi2.5-HA7.5	331.86	379.36	12.16
S11	NR/RSi2.5-HA7.5/CA1	324.71	379.71	12.32

**Table 4:** Dynamic mechanical properties of the prepared natural rubber composites.

Samples	Materials	Tg (°C)	Tan δ at 0 °C	Tan δ at 60 °C
S1	NR	−50.17	0.0471	0.0260
S2	NR/RSi10	−49.90	0.0719	0.0433
S3	NR/RSi7.5-HA2.5	−49.78	0.0685	0.0401
S4	NR/RSi5.0-HA5.0	−47.81	0.0879	0.0371
S5	NR/RSi2.5-HA7.5	−49.38	0.0549	0.0251
S6	NR/HA10	−51.32	0.0444	0.0225
S7	NR/RSi2.5-HA7.5/gDPNR5	−51.60	0.0520	0.0266
S8	NR/RSi2.5-HA7.5/gDPNR10	−50.18	0.0561	0.0261
S9	NR/RSi2.5-HA7.5/gDPNR20	−51.46	0.0539	0.0251
S10	gDPNR/RSi2.5-HA7.5	−50.71	0.0586	0.0201
S11	NR/RSi2.5-HA7.5/CA1	−50.89	0.0504	0.0276

## Data Availability

Data are contained within the article.
